# Lessons learned from an evaluation of referrals to the emergency department

**DOI:** 10.1186/s13584-020-00377-2

**Published:** 2020-04-27

**Authors:** Roee Gorodetzer, Evan Avraham Alpert, Zvika Orr, Shifra Unger, Todd Zalut

**Affiliations:** 1 Faculty of Life and Health Sciences, Jerusalem College of Technology, 21 Havaad Haleumi St, 9372115 Jerusalem, Israel; 2 Department of Emergency Medicine, Shaare Zedek Medical Center, Jerusalem, Israel

**Keywords:** Urgent care centers, Emergency departments, Hospitals, Referrals

## Abstract

**Background:**

Emergency department (ED) crowding is an international phenomenon dependent on input, throughput, and output factors. This study aims to determine whether patterns of potentially unnecessary referrals from either primary care physicians (PCPs) or urgent care centers (UCCs) can be identified, thereby to reduce ED visits by patients who could be treated elsewhere. Literature from the United States reports up to 35% unnecessary referrals from UCCs.

**Methods:**

A retrospective cohort study was conducted of patients referred to an ED in Jerusalem by either their PCP or a group of UCCs with a full range of laboratory tests and basic imaging capabilities between January 2017 and December 2017. The data were analyzed to identify referrals involving diagnoses, specialist consultations, and examinations unavailable in the PCP’s office or UCC (e.g., ultrasound, CT, echocardiogram, or stress test); these referrals were considered necessary for completion of the patient work-up. If patients were evaluated by an ED physician and sent home after an examination or laboratory test available at least in the UCC, the referrals were considered potentially unnecessary.

**Results:**

Significantly more referrals were made by PCPs than UCCs (1712 vs. 280, *p* < 0.001). Significant differences were observed for orthopedics, general surgery, and obstetrics/gynecology referrals (*p* = 0.039, *p* < 0.001, *p* = 0.003). A higher percentage of patients referred by PCPs had potentially unnecessary visits compared to patients referred by UCCs (13.9% vs. 7.9%, *p* = 0.005).

**Conclusion:**

A robust UCC system may help further reduce potentially unnecessary visits (including complex patients) to the ED.

## Introduction

When patients arrive at a crowded emergency department (ED), they are likely to experience treatment delays, longer wait times, and increased medical errors, which may result in poorer health outcomes, including death [1–4]. ED crowding also has negative effects on universally accepted professional ethical standards of patient privacy, beneficence, nonmaleficence, patient autonomy, and justice. Specifically, “crowding” is defined as a situation in which patients’ needs for emergency medical services exceed the resources available for treatment in the ED, hospital, or both [5]. It is affected by three main categories of factors: input, throughput, and output [6, 7].

### Input factors

Input factors reflect patient flow into the ED [5–7]. Factors that have been studied include nonurgent ED visits. Reasons for nonurgent ED referrals include a lack of community medical services beyond regular working hours (evenings, nights, and weekends), physician referrals, pain complaints, minor trauma, age, convenience, seasonality, and belief in the superior treatment capabilities of ED physicians. Certain characteristics of primary care are also associated with an increased likelihood of visiting the ED, including lack of a regular physician, unmet healthcare needs, poor continuity of care, and perceived lack of rapid access to care [8].

### Throughput factors

Throughput factors reflect workflow and bottlenecks within the ED [6, 7]. ED resources include staff, beds, registration, documentation, and laboratory testing. Additionally, inefficient work processes can lead to delays in treatment. These include a lack of continuity of patient care due to shift changes, communication problems between the treating teams, and lack of access to important medical information. Extra-ED factors include delays caused by waiting for laboratory tests, imaging studies, and consultants.

### Output factors

Output factors reflect bottlenecks in the discharge process or hospitalization stage [6, 7], and can be measured based on length of stay (LOS) [6]. LOS can be prolonged due to “access block,” when a patient is forced to wait in the ED for over 8 h due to lack of proper access to an inpatient hospital bed [2].

### Health system in Israel

In Israel, health insurance is mandated by the National Health Insurance Law (1994), and coverage for every citizen is ensured through four Health Maintenance Organizations (HMOs) [9]. Services include hospitalizations, primary care, specialty consultations, prescription drugs, certain preventive services, mental health care, and dental care for children [10]. Only soldiers, who receive health care directly from the military, are exempt from the system.

An integral part of Israel’s health care system is urgent care. Urgent care centers (UCCs) may be private or operated through an HMO. Each of the four HMOs maintains UCCs throughout Israel, with the Meuhedet HMO listing the most, at 100 sites (https://www.meuhedet.co.il). However, many of these centers are open only during certain hours of the day or evening. In the United States, there are over 9000 UCCs [11]. Israel’s major private UCC operator is known as TEREM (acronym in Hebrew for Immediate Medical Treatment), which currently operates 24 clinics throughout the country, many for 24 h a day (https://terem.com/Clinics.aspx?lang=1). The centers are staffed by physicians and paramedical staff, including nurses, emergency medical technicians, or paramedics. Although not all physicians at TEREM UCCs are board-certified in a specialty, the organization does arrange numerous training sessions and asynchronous learning programs to teach advanced urgent care skills and concepts. They also have an on-call specialist for phone consultations. The centers can perform advanced blood tests, including troponin and D-dimer; this means that in a subset of low-risk patients, they have the capability to rule out both a myocardial infarction and a pulmonary embolus or deep venous thrombosis. During 2012, a total of 200,000 patients visited TEREM UCCs in Jerusalem, similar to the number of visits to hospitals in Jerusalem in the same year (D. Zimmerman, personal communication, April 23, 2017). The Ministry of Health reports that cities with a TEREM UCC have 35 to 45% fewer ED visits [12].

TEREM was first established in Jerusalem, a city of 882,652 residents with three hospitals, the largest of which is the Shaare Zedek Medical Center (SZMC) (Central Bureau of Statistics, 2016). In 2014, there were 128,769 visits to the ED in Jerusalem (including adult, pediatric, and obstetric patients) with an admission rate of 34.67%. Unlike most other hospitals throughout the country, Jerusalem’s hospitals are not run by the government or a particular HMO. Rather, their capital expenses are raised through private funds*.*

### Physician referrals

Unnecessary ED referrals made by medical personnel may contribute to the input factors that cause crowding [6]. In general, patients who use a usual source of primary care receive more appropriate preventive care and experience fewer ED visits and hospitalizations [8]. In Israel, the percentage of ED visits with a medical referral is increasing, with 69% in 2015 compared to 66% in 2010 and 63% in 2006 (excluding maternity visits) [13].

Referrals to the ED can be made by the patient’s primary care physician (PCP) or any number of physicians affiliated with one of Israel’s many UCCs. Obtaining a referral is important both to reduce ED overcrowding and for financial reasons, as patients must pay 800 New Israel Shekels (approximately 218 US dollars) to receive care in an ED without a referral. It should be noted that there are several exemption cases based on the Israel National Insurance Law for which a referral is not needed. Examples include a visit that results in hospitalization, involvement in a traffic accident with a letter from the police, confirmed school injury, new fracture, shoulder dislocation, foreign body to the eye, complication of cancer, and active labor (https://www.health.gov.il/English/Services/Citizen_Services/Pages/ER.aspx).

There is a paucity of literature that evaluates why physicians decide to refer patients to the ED and whether these referrals are necessary. A recent study of ED referrals from a number of UCCs local to Las Vegas, Nevada found that 35.9% (28.8% in adults) were not justified [14].

Interestingly, there is no consensus on the definition of “unnecessary” or “unjustified” referrals. Zitek and colleagues assume they refer to visits by patients who have no advanced imaging studies, procedures, or specialty consultations done in the ED and are not admitted to the hospital [14]. In the pediatric literature, “essential ED interventions” include simple procedures such as incision and drainage, catheterization, and laceration repair [15]. Other studies imply that “unnecessary” visits are those not requiring urgent treatment or specialist input [16]. This is problematic because concerns or health issues that initially seem urgent or emergent may, in the end, be benign, but evaluation in the emergency department may still be justified. A classic example is epigastric pain in a patient over 50, which may include coronary disease in the differential diagnosis, even if the patient is ultimately discharged with reflux or gastritis. This study objectively defines an “unnecessary” ED visit as one where the referring center has the same resources as the ED, and no further studies are carried out prior to the patient’s discharge.

Finally, being referred to the hospital is not always better. Interestingly, patients diagnosed with hypertensive urgency who were treated outside of a hospital had better outcomes than those sent to the ED for the same diagnosis [17].

This study aims to determine whether patterns of potentially unnecessary referrals from either PCPs or UCCs can be identified, which could be used to reduce unnecessary inflow to the ED. The results may lead to process improvement in the healthcare system.

## Methods

A retrospective cohort study was conducted of patients referred to the general ED of SZMC by either a TEREM UCC or a PCP between January and December 2017. Relevant charts were identified via a checkbox relating to the method of referral clicked in the electronic health record during the patient’s registration. A cohort was taken of visits during the first 3 days of each consecutive month, chosen as representative of weekdays, weekends, and holidays throughout the various seasons of the year. Patients who arrived at the ED by ambulance were excluded.

The data were analyzed to identify diagnoses, specialist consultations, and examinations unavailable in the PCP’s office or the UCC (e.g., ultrasound, CT, echocardiogram, or stress test). If a patient received such a consultation or examination, the referral was considered necessary. In contrast, if a patient was evaluated in the ED and sent home after an examination or laboratory test available at least through the UCC, the referral was considered potentially unnecessary. Data were entered into Excel (Redmond, WA, USA: Microsoft) by a group of six students who participated in a training session on data abstraction as part of their statistics and research methods courses in nursing school. Each student primarily analyzed two consecutive months of data and secondarily analyzed a different student’s data for verification. Any discrepancies were reconciled by one of the two lead authors (RG or TZ). The dataset was analyzed using SPSS Statistics for Windows version 25.0 (Armonk, NY, USA: IBM Corp). The study protocol was approved by the SZMC Institutional Review Board, known as the Helsinki Committee.

## Results

### Subjects’ demographics

Subjects’ ages ranged from 2 to 99 years, with the most visits made by those between 20 and 39 (37.1%). In this group, 46.6% of visits were for obstetric/gynecological (OB/GYN) issues. Although most patients below the age of 18 were sent directly to the SZMC pediatric ED, a small number of children were seen in the general ED for orthopedic problems. This was due to convenience because at that time the general ED had a room equipped with orthopedic supplies and staffed by an orthopedic resident at all times. The majority of visitors (55.8%) were women (Table 1).

**Table 1:** Subjects’ demographics

Age Group	Number of Visits	Percent
0–19	71	3.6
20–39	739	37.1
40–59	450	22.6
60–79	478	24
80–99	254	12.8
**Men**	881	44.2
**Women**	1111	55.8
Total	1992	100

### Visits according to specialty

Emergency medicine is a relatively new specialty in Israel. During the study period, triage was based on both the Canadian Triage and Acuity Scale and various specialty services. Most patients were seen by either a resident or an attending physician in emergency or internal medicine (45.1%), but many were seen directly by a specialist. In this ED, discharge privileges are given only to residents who have finished a one-year rotating internship, have a minimum of approximately 2 years of experience in their specialty, and have passed their written boards. Therefore, even the most junior physicians entitled to discharge patients have at least 3 years of experience, the same as board-certified internists or emergency physicians in the United States. The most common specialty referrals were for general surgeons, obstetricians/gynecologists, or orthopedists. Other referrals were seen by ophthalmologists, otolaryngologists (ENTs), and urologists (Table 2). 
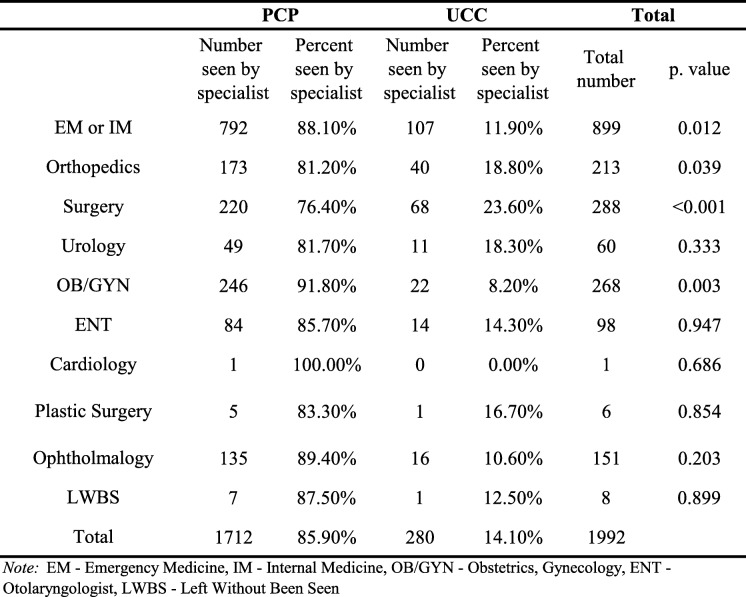

**Table 2:** Comparison of referrals between specialists

	PCP		UCC		Total	
	Number seen by specialist	Percent seen by specialist	Number seen by specialist	Percent seen by specialist	Total number	*p*. value
EM or IM	792	88.10%	107	11.90%	899	0.012
Orthopedics	173	81.20%	40	18.80%	213	0.039
Surgery	220	76.40%	68	23.60%	288	<0.001
Urology	49	81.70%	11	18.30%	60	0.333
OB/GYN	246	91.80%	22	8.20%	268	0.003
ENT	84	85.70%	14	14.30%	98	0.947
Cardiology	1	100.00%	0	0.00%	1	0.686
Plastic Surgery	5	83.30%	1	16.70%	6	0.854
Ophtholmalogy	135	89.40%	16	10.60%	151	0.203
LWBS	7	87.50%	1	12.50%	8	0.899
Total	1712	85.90%	280	14.10%	1992	

*Note: EM* Emergency Medicine, *IM* Internal Medicine, *OB/GYN* Obstetrics, Gynecology, *ENT* Otolaryngologist, *LWBS* Left Without Been Seen

Significantly more referrals were made across the board by PCPs compared to UCCs (1712 vs. 280, *p* < 0.001). This was consistent for all specialties, with statistical significance for orthopedics, general surgery, and OB/GYN cases (*p* = 0.039, *p* < 0.001, and *p* = 0.003, respectively) (Table 2).

### Necessary vs. potentially unnecessary referrals

A higher percentage of patients referred by PCPs had potentially unnecessary visits than patients referred by UCCs (13.9% vs. 7.9%, *p* = 0.005) (Table 3).

**Table 3:** Referrals by source (PCP and UCC)

	PCP		UCC		*p*. value
	Number of visits	Percent	Number of visits	Percent	
Necessary	1474	86.1	258	92.1	0.005
Unnecessary	238	13.9	22	7.9	
Total	1712	100.0	280	100.0	

*PCP* primary care physician, *UCC* urgent care center

### Referrals according to district

The city of Jerusalem officially includes 58 neighborhoods, making it difficult to calculate referrals by neighborhood. For statistical calculation and clear data segmentation, neighborhoods were grouped into districts based on geographical proximity according to a map of the Jerusalem municipality used by the Central Bureau of Statistics (https://jerusaleminstitute.org.il/wp-content/uploads/2019/05/shnaton_SA_Area_Map.png). Twenty districts were included in the analysis: 18 in the Jerusalem metropolitan area, 14 in Jerusalem itself (A-M), and 4 outside of Jerusalem. Each included several adjacent neighborhoods. One group (“Other”) was located outside of the Jerusalem metropolitan area and one (“Unknown”) represented referrals from an undetected address. District E had the highest percentage of potentially unnecessary referrals (19.70%) compared to the rest of the districts (*p* = 0.029), and a total of five districts had 15% or more potentially unnecessary referrals)Table 4). 
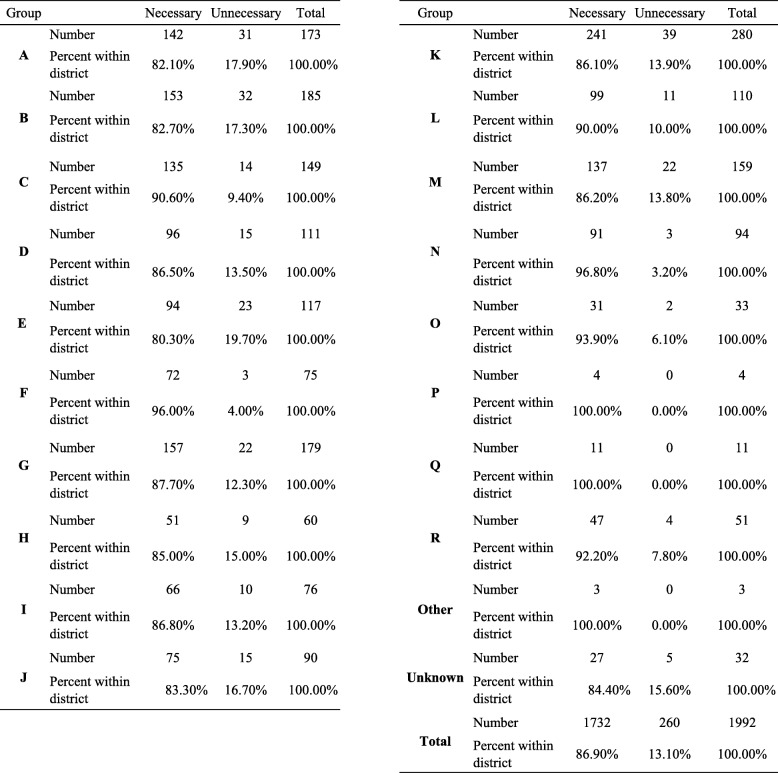

**Table 4:** Referrals according to district

Group		Necessary	Unnecessary	Total
**A**	Number	142	31	173
	Percent within district	82.10%	17.90%	100.00%
**B**	Number	153	32	185
	Percent within district	82.70%	17.30%	100.00%
**C**	Number	135	14	149
	Percent within district	90.60%	9.40%	100.00%
**D**	Number	96	15	111
	Percent within district	86.50%	13.50%	100.00%
**E**	Number	94	23	117
	Percent within district	80.30%	19.70%	100.00%
**F**	Number	72	3	75
	Percent within district	96.00%	4.00%	100.00%
**G**	Number	157	22	179
	Percent within district	87.70%	12.30%	100.00%
**H**	Number	51	9	60
	Percent within district	85.00%	15.00%	100.00%
**I**	Number	66	10	76
	Percent within district	86.80%	13.20%	100.00%
**J**	Number	75	15	90
	Percent within district	83.30%	16.70%	100.00%
**K**	Number	241	39	280
	Percent within district	86.10%	13.90%	100.00%
**L**	Number	99	11	110
	Percent within district	90.00%	10.00%	100.00%
**M**	Number	137	22	159
	Percent within district	86.20%	13.80%	100.00%
**N**	Number	91	3	94
	Percent within district	96.80%	3.20%	100.00%
**O**	Number	31	2	33
	Percent within district	93.90%	6.10%	100.00%
**P**	Number	4	0	4
	Percent within district	100.00%	0.00%	100.00%
**Q**	Number	11	0	11
	Percent within district	100.00%	0.00%	100.00%
**R**	Number	47	4	51
	Percent within district	92.20%	7.80%	100.00%
**Other**	Number	3	0	3
	Percent within district	100.00%	0.00%	100.00%
**Unknown**	Number	27	5	32
	Percent within district	84.40%	15.60%	100.00%
**Total**	Number	1732	260	1992
	Percent within district	86.90%	13.10%	100.00%

Legend of districts: Districts within Jerusalem itself: A - At-Tur, Silwan, Isawiya, Wadi al-Joz. B - Kfar Aqab, Atarot, Beit Hanina, Shuafat. C - Tzur Baher, Abu Tor, Umm Tuba, Jabel Mukaber, Beit safafa. D - Neve Yaakov, Pisgat Zeev. E - Ramat Shlomo, Ramot. F - Givaa Tzarfatit, Ramot Eshkol, Shmuel Hanavi. G - Buharim, Beit David, Lev hair, Geula, Mea shearim, Old City, Rehavia. H - Romema, Kiryat Haleom. I - givat Shaul, Har Nof, Kiryat Moshe. J - Beit Hakerem, Yefe Nof, Givat Mordechai. K - Emek Refaim, Baka, Gonenim, Har Homa, Talpiyot, Armon Hanaziv. L – Gilo. M - Kiryat Yovel, Kiryat Menahem, Bait Vegan, Malha. Districts outside of Jerusalem: N - Beit Shemesh. O - Gush Etzion. P – Binyamin. Q - Mevaseret Zion, Beit Zayit, Moza, Abu Ghosh. R - Maale adumim and surrounding area. Other – not from the area of Jerusalem metropolin. Unknown - undetected address

## Discussion

Significantly more referrals were made by PCPs than UCCs (1712 vs. 280, *p*-value < 0.001). This was consistent across all specialty groups. Additionally, significantly more patients referred by PCPs had potentially unnecessary visits, compared to patients referred by UCCs (13.9% vs. 7.9%, *p* = 0.05). This may be expected, as PCP offices have fewer resources than UCCs. However, although PCPs made six times as many referrals as UCCs, the absolute difference in potentially unnecessary referrals between the two was only six percentage points. This can be explained by the overall strong emphasis in PCP clinics on appropriate referrals to the ED. It would be interesting to examine whether a certain percentage of patients referred to the ED by PCPs could instead be seen and appropriately treated in UCCs.

The rates of potentially unnecessary referrals from both PCPs and UCCs were much lower than the recently published results for the single-center study in Las Vegas, which found that 35.9% of referrals (28.8% for adults only) from surrounding UCCs were inappropriate [14]. This appears to be due to the capabilities of the different centers in Las Vegas. Almost all centers were able to perform electrocardiograms and X-rays, but only 79.6% could perform some type of blood test, and only 59.3% had a physician present at all times. A large percent of referrals for chest pain (43.8%) were deemed to be unnecessary. The most likely explanation for the differences between these studies’ results is that the UCCs in the Jerusalem area were run by a single organization (TEREM) and all had the ability to perform advanced laboratory tests and radiographs. They also conduct numerous training sessions emphasizing issues in urgent care. A clear lesson is that a robust system of UCCs surrounding a tertiary care hospital may help reduce unnecessary referrals, although more work must be done to reduce this number further.

It should be noted that reducing unnecessary referrals is not the only solution for ED overcrowding, which is a complex and multi-system issue. Additional solutions within the ED itself include point-of-care testing, nurse-requested X-rays, alternative short-stay units, team triage, and improved patient streaming [18]. Telemedicine, which has traditionally been used to diagnose and treat patients in remote areas, has also been used in attempts to reduce overcrowding by screening patients with lower triage scores [19, 20] and treating patients in the community to prevent their arrival at the ED [21]. System-wide responses include improving access to primary care [22], post-acute care beds [23], and reducing inflow to overcrowded EDs through ambulance diversion [24].

Interestingly, although one might expect to see the elderly age group at the top of the list, the most referrals to the ED were for patients aged 20 to 39. Similarly, according to the Ministry of Health, patients between the ages of 22 and 34 accounted for 764,800 visits in 2016, whereas patients age 65 and above accounted for only 564,500. This may be partially explained by a large number of OB/GYN visits in the younger age group. Additional explanations could be that when it comes to elderly patients, either community healthcare seeks to keep them out of the hospital, or (probably more likely) that elderly patients go directly to the hospital because they believe they are more ill. In the United States, patients aged 75 and older account for the largest number of ED visits compared to other age groups [25]. A similar trend is seen in Israel in that the rate of nonmaternity visits to the ED is highest in patients over age 75 [26].

There were significant differences in ED referrals by district, with five districts having over 15% potentially unnecessary referrals. An in-depth analysis of the UCCs close to those districts is needed to determine whether they provide the same quality of care as the others. The differences may also be linked to characteristics of the population served or their ability to get to the hospital.

Direct educational interventions may reduce unnecessary ED visits. Educational interventions have proven useful in a number of different medical settings. For example, educational interventions for adults who visit the ED with suspected or diagnosed asthma have been shown to decrease subsequent hospital admissions [27]. Interventions using text messaging have been used to improve outcomes in patients with alcohol abuse [28] and to help patients self-manage long-term illnesses [29].

Communication between EDs and PCPs is essential [30]. One method of fostering this education and communication would be to send general information on reducing unnecessary referrals to all physicians in the relevant districts. Another would be to send a link to the specific referring physician in every case where the ED visit was felt to be unnecessary.

Overall, a low number (< 1%) of patients left without being seen. These referrals were defined as unnecessary (although this was uncertain). Such small numbers would not have skewed the data in any direction and would not have changed the overall conclusions of this study. According to previous studies, patients may have left without being seen because PCPs were trying to limit their office or clinic schedules, or referrals were given following patients’ complaints over the phone [31].

Although a previous study has shown that low-complexity patients have a negligible effect on LOS, these were all patients with a Canadian Triage and Acuity Score of 4 or 5 [32]. Additionally, a recent study of referrals of UCC patients to a single tertiary care ED in Pennsylvania found that 80.1% were categorized as complex. However, many of the tests and procedures defined as complex are routinely performed in TEREM UCCs, including X-rays, IV placement, incision and drainage, foreign body removal, and complex laceration repair [33]. In addition to these tests and procedures, TEREM UCC physicians routinely evaluate and discharge large numbers of higher acuity patients, including those with chest pain and shortness of breath. Perhaps further reducing numbers of “complex” patients such as these could further minimize ED crowding.

### Limitations

Because this study involved a single center in an urban center of Israel, the results may not be generalizable to other hospitals in Israel or around the world. The UCCs surrounding the target hospital in this study may have both more resources and higher quality staff than those in other cities or countries. Additionally, although more unnecessary visits were referred by PCPs than UCCs, the study did not examine how many of the patients who were referred to the ED from the UCC were previously sent to the UCC by their PCP.

The definition of a potentially unnecessary visit is not standardized in the medical literature. Although this study assumes that the emergency department is the “gold standard” of care, there was no outcome data for any of the patients who were discharged. However, the SZMC does have a rigorous auditing process, and it is expected that there would have been feedback in the case of a negative outcome. Further analysis of the breakdown of cases by surgical subspecialty is also necessary to determine whether further training of UCC physicians could reduce unnecessary referrals.

## Conclusion

Whereas the current trend in ED management is to attempt to improve throughput and output, an emphasis on reducing unnecessary referrals may also help reduce crowding, especially in environments with robust and highly developed UCCs.

A system of UCCs staffed by physicians and capable of performing a full range of laboratory tests and basic imaging studies in the area surrounding EDs may reduce unnecessary visits. This may be especially true as many of these potentially unnecessary visits are more complex than those previously described in the literature. It is worth investigating whether feedback and educational interventions may help to further reduce the number of unnecessary visits.

## Data Availability

The datasets used and/or analyzed during the current study are available from the corresponding author on reasonable request.

## Abbreviations

ED: Emergency Department
PCP: Primary Care Physician
(UCC): Urgent Care Center
LOS: Length of Stay
SZMC: Shaare Zedek Medical Center
HMO: Health Maintenance Organization
RG: Roee Gorodetzer
TZ: Todd Zalut
OB/GYN: Obstetrics/Gynecology
ENTs: Otolaryngologists
